# Drug-primed reinstatement of cocaine seeking in mice: increased excitability of mediumsized spiny neurons in the nucleus accumbens

**Published:** 2013-10-25

**Authors:** Yao-Ying Ma, Sandy M. Henley, Jeff Toll, James D. Jentsch, Christopher J. Evans, Michael S. Levine, Carlos Cepeda

Volume 5(4) 2013, pp. 257–271, e00122

The funding section in this paper was incomplete. The correct funding information appears below.

